# Supplementary material for: Biological Significance of the Protein Changes Occurring in the Cerebrospinal Fluid of Alzheimer’s Disease Patients: Getting Clues from Proteomic Studies
Source: Diagnostics (Basel). 2021 Sep 9;11(9):1655. doi: 10.3390/diagnostics11091655 (PMC8467255; doi:10.3390/diagnostics11091655)
Supplement: Supplementary file 1 [file diagnostics-11-01655-s001.zip › Supplemental Table S2.pptx]

## Slide 1
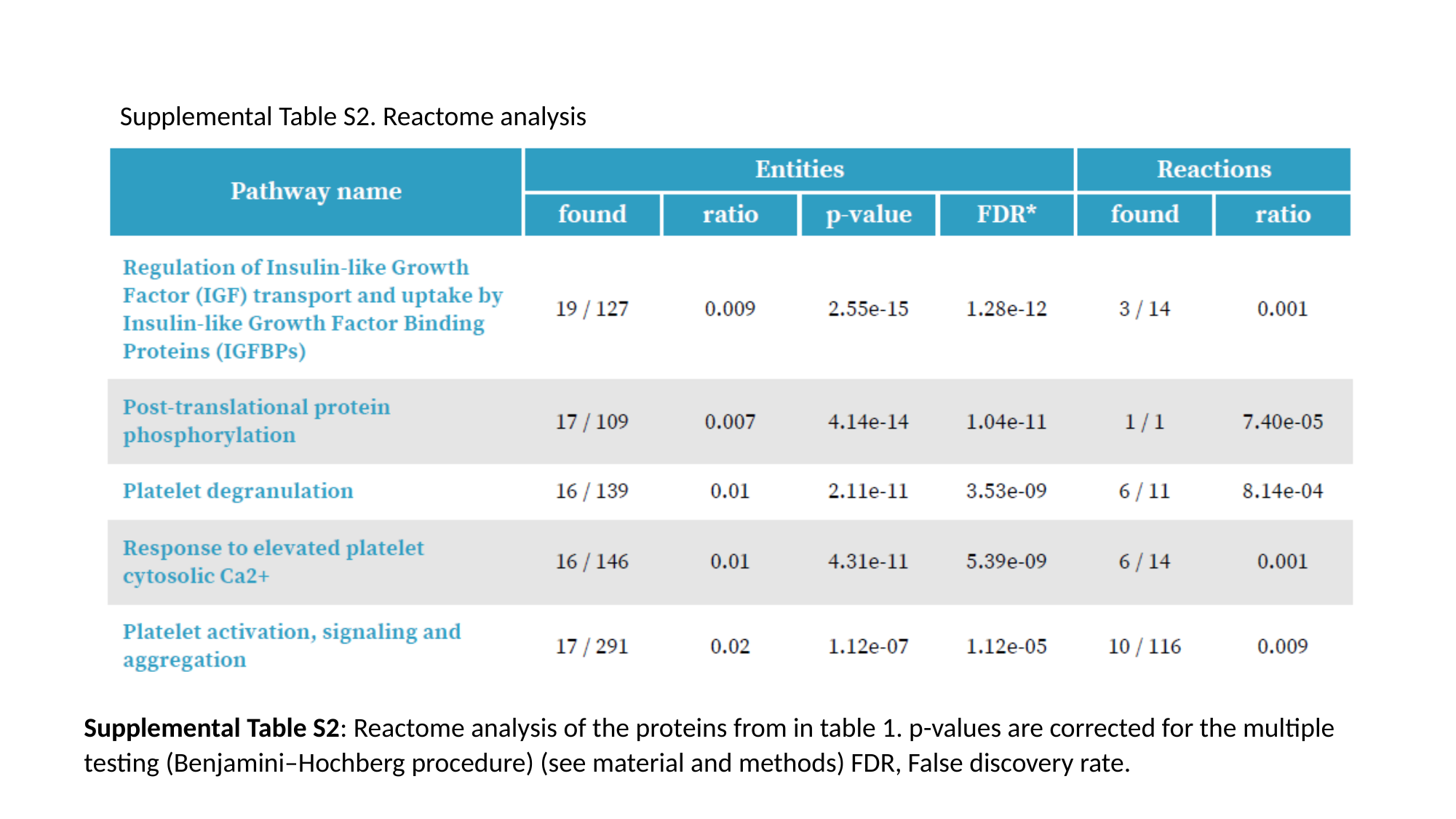

Supplemental Table S2. Reactome analysis
Supplemental Table S2: Reactome analysis of the proteins from in table 1. p-values are corrected for the multiple testing (Benjamini–Hochberg procedure) (see material and methods) FDR, False discovery rate.
